# A newly defined risk signature, consisting of three m^6^A RNA methylation regulators, predicts the prognosis of ovarian cancer

**DOI:** 10.18632/aging.103811

**Published:** 2020-09-20

**Authors:** Lili Fan, Ying Lin, Han Lei, Guang Shu, Liuer He, Zhipeng Yan, Hai Rihan, Gang Yin

**Affiliations:** 1 Department of Pathology, Xiangya Hospital, School of Basic Medical Sciences, Central South University, Changsha, Hunan Province, China; 2 Department of Immunology, School of Basic Medical Sciences, Central South University, Changsha, Hunan Province, China; 3 School of Basic Medical Sciences, Central South University, Changsha, Hunan Province, China; 4 Hunan Cancer Hospital, the Affiliated Cancer Hospital of Xiangya School of Medicine, Central South University, Changsha, Hunan Province, China

**Keywords:** RNA modification, m^6^A RNA methylation regulators, ovarian cancer, prognostic signature, risk signature

## Abstract

N6-methyladenosine (m^6^A) RNA methylation, involved in cancer initiation and progression, is dynamically regulated by the m^6^A RNA methylation regulators. However, the expression of m^6^A RNA methylation regulators in ovarian cancer and their correlation with prognosis remain elusive. Here, we demonstrated that the 18 central m^6^A RNA methylation regulators were expressed differently between ovarian cancer (OC) and normal tissues. By applying consensus clustering, all ovarian cancer patient cases can be divided into three subgroups (cluster1/2/3) based on overall expression levels of all 18 m^6^A RNA methylation regulators. We systematically analyzed the prognostic value of transcription levels of 18 m^6^A RNA methylation regulators in ovarian cancer and found that insulin-like growth factor 2 mRNA binding protein 1 (IGF2BP1), vir like m^6^A methyltransferase associated (VIRMA), and zinc finger CCCH-type containing 13 (ZC3H13) yield the highest scores for predicting the prognosis of ovarian cancer. Accordingly, we derived a risk signature consisting of transcription levels of these three selected m^6^A RNA methylation regulators as an independent prognostic marker for OC and validated our findings with data derived from a different ovarian cancer cohort. Moreover, by the Gene Set Enrichment Analysis (GSEA), we demonstrated that the three selected regulators were all correlated with pathways in cancer and WNT signaling pathways. In conclusion, m^6^A RNA methylation regulators are vital participants in ovarian cancer pathology; and IGF2BP1, VIRMA, and ZC3H13 mRNA levels are valuable factors for prognosis prediction and treatment strategy development.

## INTRODUCTION

Ovarian cancer (OC) accounts for 2.5% of all malignancies among females, but for 5% of all cancer deaths due to its relatively high fatality rate, as about 80% of patients are diagnosed with the advanced disease [1]. At the time of diagnosis, most of the OC had metastasized to the uterus, bilateral appendage, omentum, and pelvic organs. With recent advances in surgery, chemotherapy, and novel immunotherapy, the overall survival of OC at every stage has been improved. However, there is still a lack of reliable prognostic indicators for OC.

Although the multiple layers of epigenetic regulation, such as modification of DNA and proteins, have been identified, the mechanism of RNA modification and its role in OC pathology remains unclear. Several common RNA modification forms have been documented including N6-methyladenosine (m^6^A), N1-methyladenosine (m^1^A) and 5-methylcytosine (m^5^C) [2, 3]. As the most bountiful internal modification on mRNA, m^6^A occurs on adenosine and is enriched near stop codon and 3’ untranslated terminal region [4, 5]. Similar to DNA modification, the m^6^A RNA methylation is dynamically regulated by the m^6^A RNA methylation regulating elements, which are called “writers”, “erasers”, or “readers” depending on distinct functions. “Writers” introduce a methyl group on A of RRACH sequence (R = A or G, H = A, C or U) near the stop codon, 3’ untranslated region (UTR) and long internal exon through the methyltransferase complex (MTC) consisting of METTL3 as the core component and other related subunits including METTL14, WTAP, VIRMA, RBM15 and ZC3H13 [3, 6]. “Erasers” include FTO and ALKBH5, which mediate the demethylation reaction. These two layers of regulations make m^6^A a dynamic and reversible process. “Readers” are a group of RNA binding proteins that recognize the m^6^A methylation and perform corresponding functions. These proteins include YT521-B homology (YTH) domain-containing proteins, eukaryotic initiation factor 3 (eIF3), insulin-like growth factor 2 mRNA-binding proteins (IGF2BPs) and heterogeneous nuclear ribonucleoprotein (HNRNP) protein family members. Interestingly, recent studies have shown that the “writers” can also participate in post-methylation-regulation on target RNA and thereby partially function as “readers” [7, 8]. The m^6^A modification regulates a variety of critical steps in the RNA life cycle starting from transcription to degradation (such as transcription, splicing, exportation, translation, and degradation) and can influence cell process (such as cell cycle progression, cell proliferation, cell apoptosis, cell migration and invasion and cell differentiation) and physiological function (such as neural development, embryonic development and adipogenesis, etc.) through modulating the life cycle of target RNA [9–14].

Experimental evidence shows that m^6^A participates in cancer pathogenesis and development [15–17]. In OC, the expression of METTL3 is elevated and associated with poor patient survival. METTL3 enhances oncogene AXL expression, resulting in the promotion of epithelial to mesenchymal transition (EMT) [18, 19]. Moreover, IGF2BP1 stabilizes the mRNA of SRF to promote its expression, leading to enhanced expression of oncogene FOXK1 and PDLIM7 in tumor cells, and a more aggressive phenotype [20]. During the treatment of OC, elevated m^6^A level contributes to resistance to poly ADP-ribose polymerase inhibitors (PARPi) through upregulating the WNT signaling pathway via enhancing the stability of FZD10. Therefore, restraining the WNT signaling pathway in combination with PARPi represents a potential therapeutic strategy for OC [21]. Despite the accumulating data indicating vital roles of m^6^A in controlling physiological and pathological processes, our knowledge about the role of m^6^A in OC oncogenesis and prognosis is far from complete.

**Figure 1:**
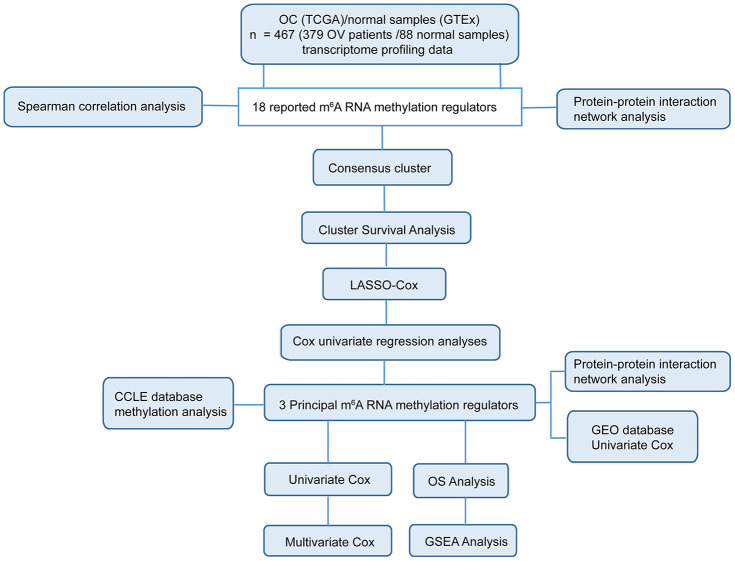
**Workflow chart of data generation and analysis.** The study mainly incorporated two sections: comprehensive bioinformatics analysis in 18 m^6^A RNA methylation regulators (including Spearman correlation analysis, protein-protein interaction analysis, consensus cluster analysis, cluster survival analysis and so on) and in the three selected m^6^A RNA methylation regulators (including CCLE database methylation analysis, protein-protein interaction network analysis, univariate Cox, multivariate Cox and so on).

The multiomics-based comprehensive analysis provides much more informative results in evaluating the expression and function of genes. In this study, we systematically analyzed the expression of 18 central m^6^A RNA methylation regulators in 379 OC with RNA sequencing data from The Cancer Genome Atlas (TCGA) datasets and in 88 normal samples with RNA sequencing data from the Genotype-Tissue Expression (GTEx) datasets. We aimed to evaluate the power of m^6^A RNA methylation regulators in predicting the prognosis of OC patients, and explore possible signaling pathways regulated by m^6^A RNA regulators in OC through comprehensive bioinformatical analysis (Figure 1). Our results indicated that the expressions of three m^6^A RNA methylation regulators, IGF2BP1, VIRMA, and ZC3H13, have strong power in predicting the prognosis of OC. We also built a risk signature gene set including these three selected m^6^A RNA methylation regulators and validated that the expression of this signature is highly correlated with the bad prognosis of OC patients using data derived from a different cohort.

## RESULTS

### The different expression of 18 m^6^A RNA methylation regulators in normal ovarian and OC tissues

**Figure 2:**
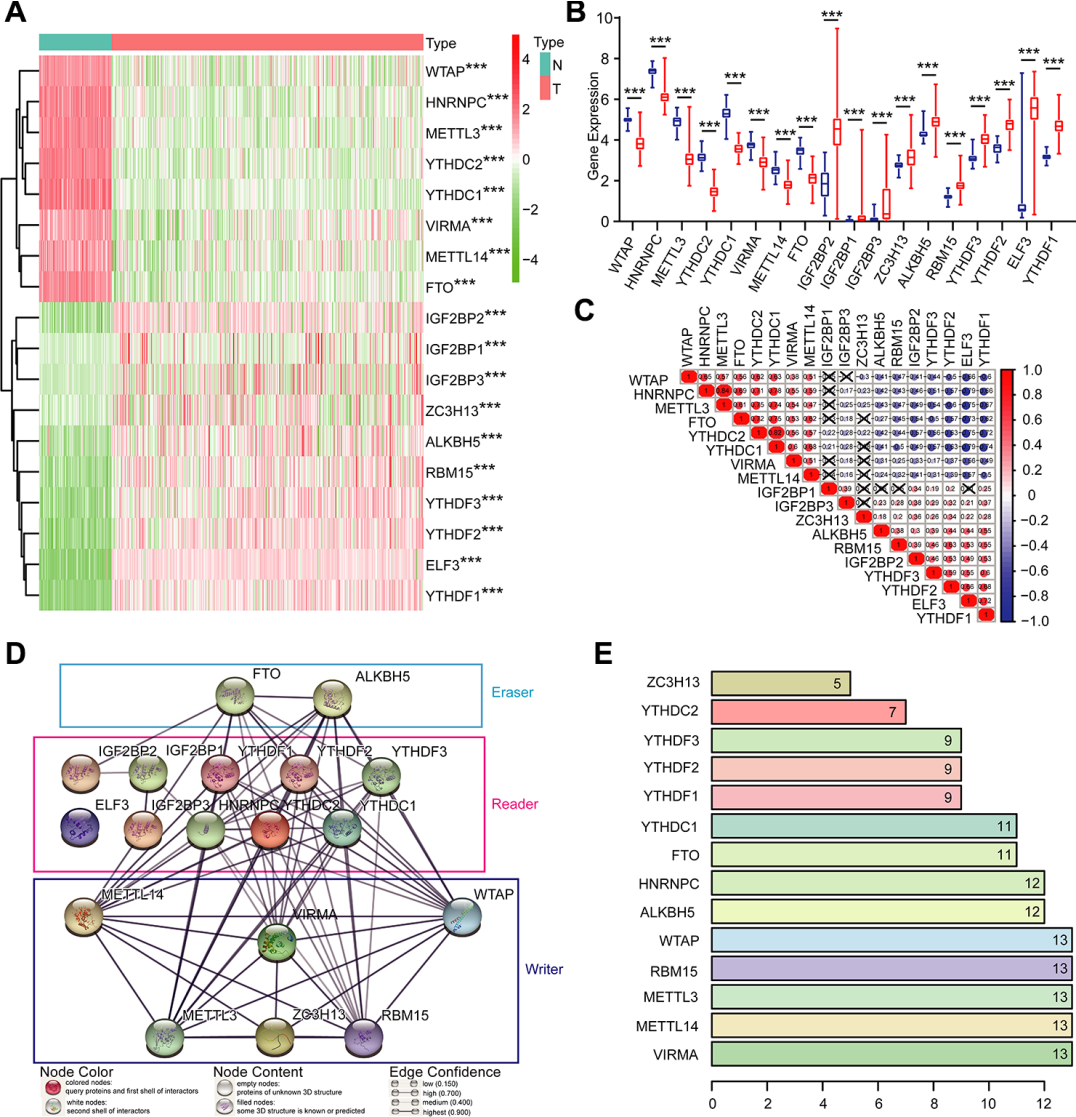
**Expression of m^6^A RNA methylation regulators and interaction among them.** (**A**) The expression levels of 18 m^6^A RNA methylation regulators in normal controls (n = 88) and OC (n = 379) with agglomerative hierarchical clustering. (**B**) Box line diagram of 18 m^6^A RNA methylation regulators. (**C**) Spearman correlation analysis of the 18 m^6^A modification regulators. (**D**) The m^6^A modification-related interactions among the 18 m^6^A RNA methylation regulators. (**E**) Number of related nodes of m^6^A RNA methylation regulators (only show the number > 5).

Given the critical functions of m^6^A RNA methylation regulators in tumorigenesis and development, we comprehensively explored the transcription of the 18 m^6^A RNA methylation regulators using the TCGA dataset. The RNA levels of m^6^A RNA methylation regulators were presented as heatmaps and box line diagrams (Figure 2A and 2B), which showed that the expression levels of m^6^A RNA methylation regulators in OC patients were significantly different from those of the normal controls. Based on the expression pattern, m^6^A RNA methylation regulators can be divided into two groups. One group (including IGF2BP2, IGF2BP1, IGF2BP3, ZC3H13, ALKBH5, RBM15, YTHDF3, YTHDF2, ELF3, and YTHDF1) is highly expressed in tumors, while the other (including WTAP, HNRNPC, METTL3, YTHDC3, YTHDC2, YTHDC1, VIRMA, METTL14, and FTO) is enriched in normal tissues.

To better understand the interactions among the 18 m^6^A RNA methylation regulators, we also inspected the correlation (Figure 2C) and interaction (Figure 2D and 2E) among these regulators. The protein-protein interaction network analysis indicated that WTAP, RBM15, METTL3, METTL14, and VIRMA have more connections with other regulators, while IGF2BP2, IGF2BP1, IGF2BP3 and ELF3 have less interaction with others (Figure 2D and 2E). Among all the “writer” genes, each gene, except for ZC3H13, has the same number of related nodes of m^6^A RNA methylation regulators (Figure 2D and 2E). The expression of ZC3H13 was also positively correlated with the “writer” gene RBM15 in OC, and negatively correlated with WTAP and METTL3, but no correlation with METTL14 and VIRMA (Figure 2C). Interestingly, the least interacting m^6^A RNA methylation regulators (ELF3, IGF2BP2, IGF2BP1, and IGF2BP3) are all “readers” (Figure 2C). We also noticed that FTO was predicted to interact with ALKBH5 (Figure 2D), and their expression levels were negatively correlated with each other in OC (Figure 2C). In addition, we further analyzed the experimentally determined interactions between these 18 m^6^A RNA methylation regulators and other proteins (Supplementary Figure 1A). It was clear that among the 18 m^6^A RNA methylation regulators, ZC3H13 had the most interactions with other proteins, mainly interacted with the cyclin-dependent kinases (CDKs) family, RNA polymerase II (POLR2) family and mediator (MED) complex family. Altogether, we concluded that most, but not all, of the 18 m^6^A RNA methylation regulators are closely associated with each other. Among them, ZC3H13 interacted mostly with CDKs, POLR2 and MED. Moreover, we also found that the frequencies of genetic changes (mutation or copy number change) of these 18 m^6^A RNA methylation regulators were relatively high (the maximum was 27%) in OC tissue compared with normal (Supplementary Figure 2), which might explain the altered expression of these genes in OC.

### Consensus clustering of m^6^A RNA methylation regulators identified three clusters of OC

**Figure 3:**
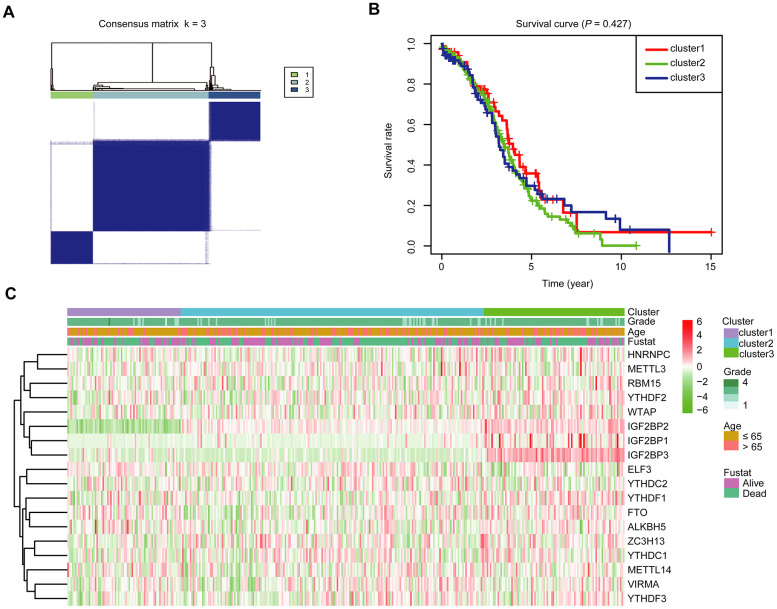
**Divergent clinicopathological features and OS of OC in the cluster1/2/3 subgroups.** (**A**) Consensus clustering matrix for k = 3. (**B**) Kaplan-Meier OS curves for 379 OC patients. (**C**) Heatmap and clinicopathologic characters of the three clusters (cluster1/2/3) defined by the m^6^A RNA methylation regulators’ consensus expression.

To further investigate the association between the expression profile of m^6^A RNA methylation regulators and the prognosis of these OC cases, we then focused on grouping all 379 OC cases according to the expression of all the 18 m^6^A RNA methylation regulators in an unbiased way through consensus clustering. With clustering stability increasing from k = 2 to 10 in the TCGA datasets, k = 3 seemed to be an acceptable selection based on the expression similarity of m^6^A RNA methylation regulators (Supplementary Figure 3A, 3B and Figure 3A). We then applied principal component analysis (PCA) to compare the transcriptional profile among cluster1, 2 and 3 groups. However, the results didn’t show a clear separation among them (Supplementary Figure 3C). Moreover, we observed that there was little difference in the overall survival (OS) rate among cluster1, 2 and 3 (Figure 3B). We further compared the clinicopathological characters of these three subgroups and found little difference among them (Figure 3C).

### Development of a risk signature consisting of three m^6^A RNA methylation regulators

**Figure 4:**
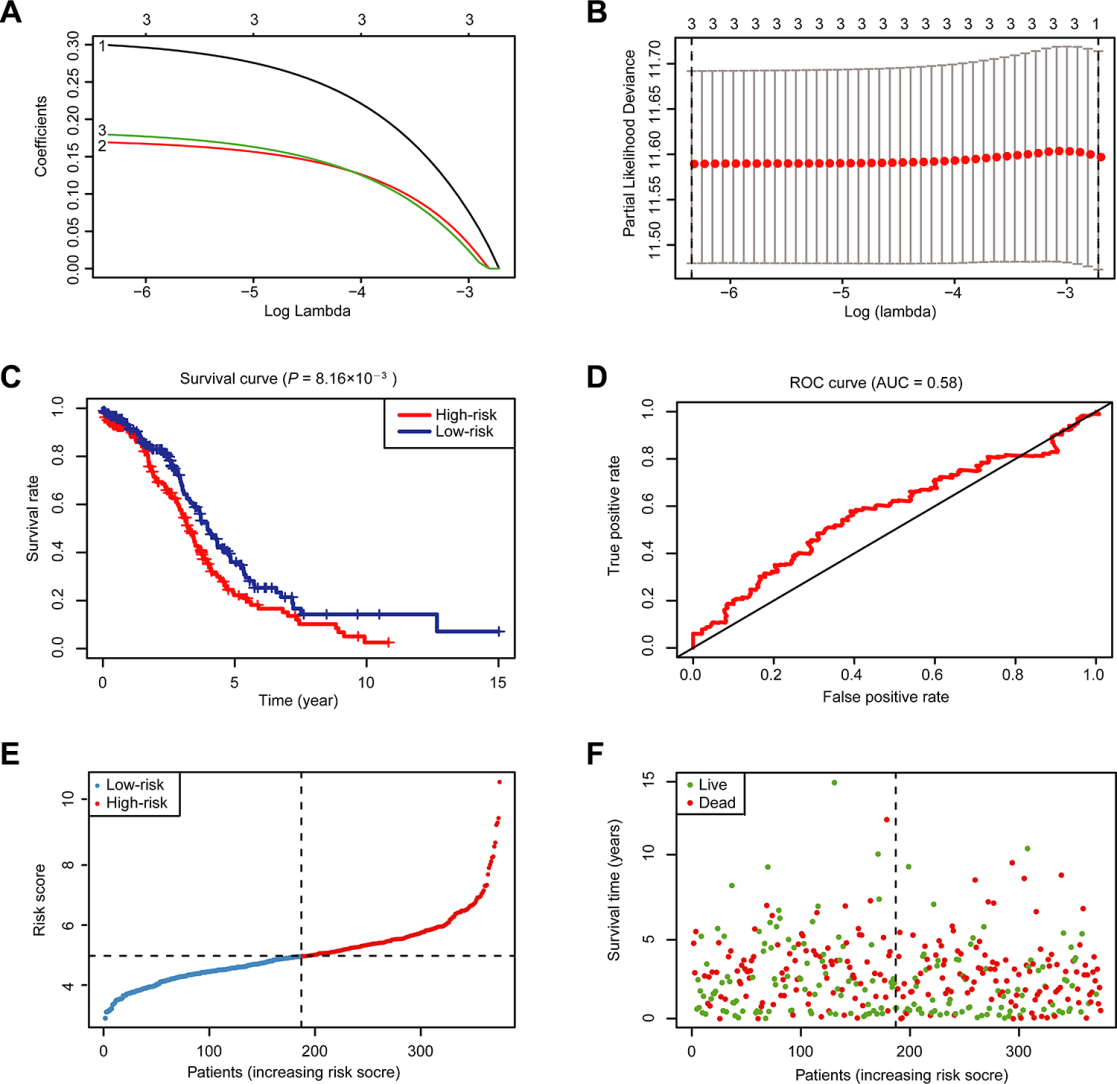
**Risk signature with 18 m^6^A RNA methylation regulators.** (**A**) LASSO regression analysis of the 18 m^6^A RNA methylation regulators. (**B**) Tenfold cross-validation for tuning the parameter selection in the LASSO regression. The solid vertical lines indicate the partial likelihood deviance with standard error. The dotted vertical lines represent the optimal values of the tuning parameter (λ) by minimum criteria. (**C**) Kaplan-Meier OS curves for patients in the TCGA datasets designated to high- and low-risk groups depended on the risk score. (**D**) ROC curves demonstrated the predictive efficiency of the risk signature in OC of TCGA datasets. (**E**–**F**) Risk score and survival status for each patient.

To better predict the clinical outcomes of OC with abnormal expression of m^6^A RNA methylation regulators, we engaged the least absolute shrinkage and selection operator (LASSO) Cox regression algorithm to the 18 regulators in the TCGA dataset, and obtained risk score through R packages LASSO regression analysis (Figure 4A, 4B). With the median risk score (median risk score = 5) as the cut-off point, we divided all patients into two groups and scrutinized notable differences in OS between the two groups (*P* < 0.05; Figure 4C). At the same time, we also examined the effect of different grades of OC patients on the prognosis. However, no statistical significance was found (Supplementary Figure 4). The receiver operating characteristic (ROC) analysis was used for testing if the survival prediction is sensitive and specific based on the risk score. The calculation of the area under the curve (AUC) values was carried out according to ROC curves (Figure 4D). And the ROC curve shows that our risk model yields supporting results as the AUC = 0.58. Hence, we compared the clinicopathological characters (including tumor pathological grade and age) of these two categories clustered by risk score (Supplementary Figure 5). We found that patients in the high-risk cluster and the low-risk cluster did not differ significantly in tumor pathological grades and age. Then, the distribution of risk score and survival status were also analyzed (Figure 4E, 4F). In Figure 4E, the risk score of each patient was arranged from low to high. Patients were divided into the low risk-group (blue dot) and the high-risk group (red dot) with the median risk score. From Figure 4F, we can see that the number of deaths of patients with high-risk scores is slightly larger than that of patients with low-risk scores.

**Figure 5:**
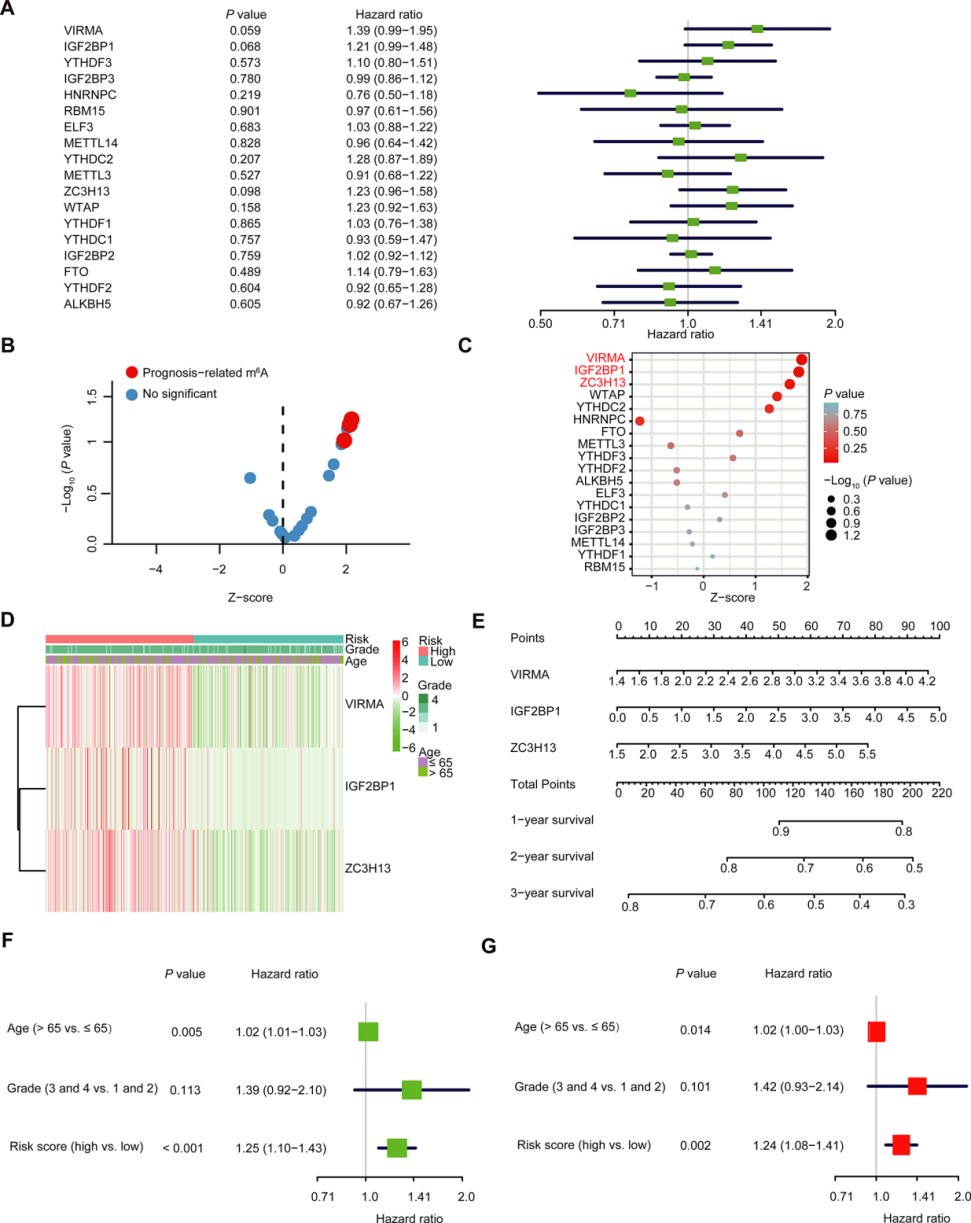
**The selection of three m^6^A RNA methylation regulators, and their effect on OC prognosis and clinicopathological characteristics.** (**A**) Cox univariate regression analyses were used to examine the associations between expression of 18 m^6^A RNA methylation regulators and prognosis. (**B**, **C**) The *P*-value of IGF2BP1, VIRMA and ZC3H13 < 0.1. (**D**) Heatmap and clinicopathologic features of the three selected m^6^A RNA methylation regulators. (**E**) Nomogram for forecasting 1-year, 2-year and 3-year survival of clinically OC patients. The nomogram is used by adding up the points identified on the points scale for each variable. Based on the sum of these points projected on the bottom scales, it is used to predict the likelihood of individual patients surviving for 1-year, 2-year and 3-year. (**F**) Univariate analysis of the hazard ratios for risk score as independent prognostic elements to anticipate the OS. (**G**) Multivariate analysis of the hazard ratios for risk score as independent prognostic elements to predict the OS.

We further explored the prognostic effect of individual m^6^A RNA methylation regulator in OC. We executed a univariate Cox regression analysis on the expression level of each m^6^A RNA methylation regulator in the TCGA dataset (Figure 5A). The results demonstrated that three out of 18 tested regulators were significantly correlated with OS (*P* < 0.1; Figure 5A–5C). These three genes (IGF2BP1, VIRMA, and ZC3H13) were all risky genes with HR > 1. Hence, we compared the clinicopathological characters (including tumor pathological grade and age) of the three regulators. It was clear that the expressions of the three selected regulators were all high in most high-risk patients (Figure 5D). To implement a quantitative method for superior outcome prediction, we established a nomogram that assimilated the three selected regulators associated with OC prognosis (Figure 5E). In this nomogram, a higher total point indicates a worse survival. Moreover, univariate and multivariate analyses for OS were executed to appraise whether clinicopathological characters (including age, stage and risk score) were independent prognostic factors of patient outcomes. Univariate analysis applying the Cox proportional hazards model for all variables demonstrated that risk score (*P* < 0.001, 95%CI HR 1.10-1.43) and age (*P* = 0.005, 95%CI HR 1.01-1.03) were all independent poor prognostic factors for OC patients (Figure 5F). Multivariate analysis applying the same variables as in the univariate analysis in the cohort supported that risk score (*P* = 0.002, 95%CI HR 1.08-1.41) and age (*P* = 0.014, 95%CI HR 1.00-1.03) were independent poor prognostic factors for OC patients (Figure 5G), but little meaningful association of OS was discovered with grade. Because different types of OC have different characteristics, and the OC samples in the TCGA database are all ovarian serous cystadenocarcinoma (OV), so we analyzed the expression of IGF2BP1, VIRMA and ZC3H13 in different types of OC in the Oncomine database (https://www.oncomine.org) (Supplementary Figure 6A–6C). The expression of IGF2BP1 in ovarian serous adenocarcinoma was higher than that in normal tissues; among ovarian clear cell adenocarcinoma, ovarian endometrioid adenocarcinoma, ovarian mucinous adenocarcinoma and ovarian serous adenocarcinoma, the expression of VIRMA was the highest in ovarian serous adenocarcinoma, and ZC3H13 was the highest in ovarian mucinous adenocarcinoma. In addition, we analyzed the expression profile of IGF2BP1, VIRMA and ZC3H13 in OC patients with different ages and tumor grades in the TCGA database on the UALCAN website (http://ualcan.path.uab.edu) [22] (Supplementary Figure 6D–6I). The expression of IGF2BP1 and ZC3H13 increased with the age of patients, while the expression of VIRMA in different age groups had no similar rule. Since there is only one sample in grade 1 and grade 4, we pay attention to the difference between grade 2 and grade 3. The expression of VIRMA in grade 3 is higher than that in grade 2, and there is statistical significance, while there is no significant difference in the expression of IGF2BP1 and ZC3H13 between grade 2 and grade 3. Moreover, the three selected m^6^A RNA methylation regulators also exist in other tumors, at least in many cell lines (Supplementary Figure 7). The mutation sites of the three selected m^6^A RNA methylation regulators in different cell lines have been shown in Supplementary Figure 7. This indicates that the m^6^A RNA methylation regulators may also play a role in other tumors too and have a certain correlation with prognosis, which needs further analysis. In particular, we also further explored the experimentally determined interactions between these three selected m^6^A RNA methylation regulators and the other proteins (Supplementary Figure 1B). Of interest, it shows that ZC3H13 interacts with the CDKs family, POLR2 family and MED complex family closely, which is consistent with the result in Supplementary Figure 1A, but IGF2BP1 had no interaction with any other proteins.

To further understand the biology of the three genes, we analyzed the protein expression of IGF2BP1, VIRMA and ZC3H13 in OC in the Clinical Proteomic Tumor Analysis Consortium (CPTAC) samples [18] (Supplementary Figure 8). The results suggest that the mRNA and protein levels of ZC3H13 are both high in cancer tissues compared with the normal control (Figure 2A and Supplementary Figure 8C). However, the protein expression of IGF2BP1 and VIRMA in the normal control group and tumor group was not consistent with the mRNA expression levels (Figure 2A and Supplementary Figure 8A–8B). This suggests that the mRNA may serve as a reservation. Only under certain circumstances, such as hypoxia or immune stimulation, can the protein be translated. A similar mode of action could be observed in the production of some cytokines. Besides, the mRNA may play a role in regulating the expression of other proteins by generating microRNAs. The specific details need further experiments and analysis.

### Validation of the risk signature using data collected from a different OC cohort, and the exploration of signaling pathways that they involve

**Figure 6:**
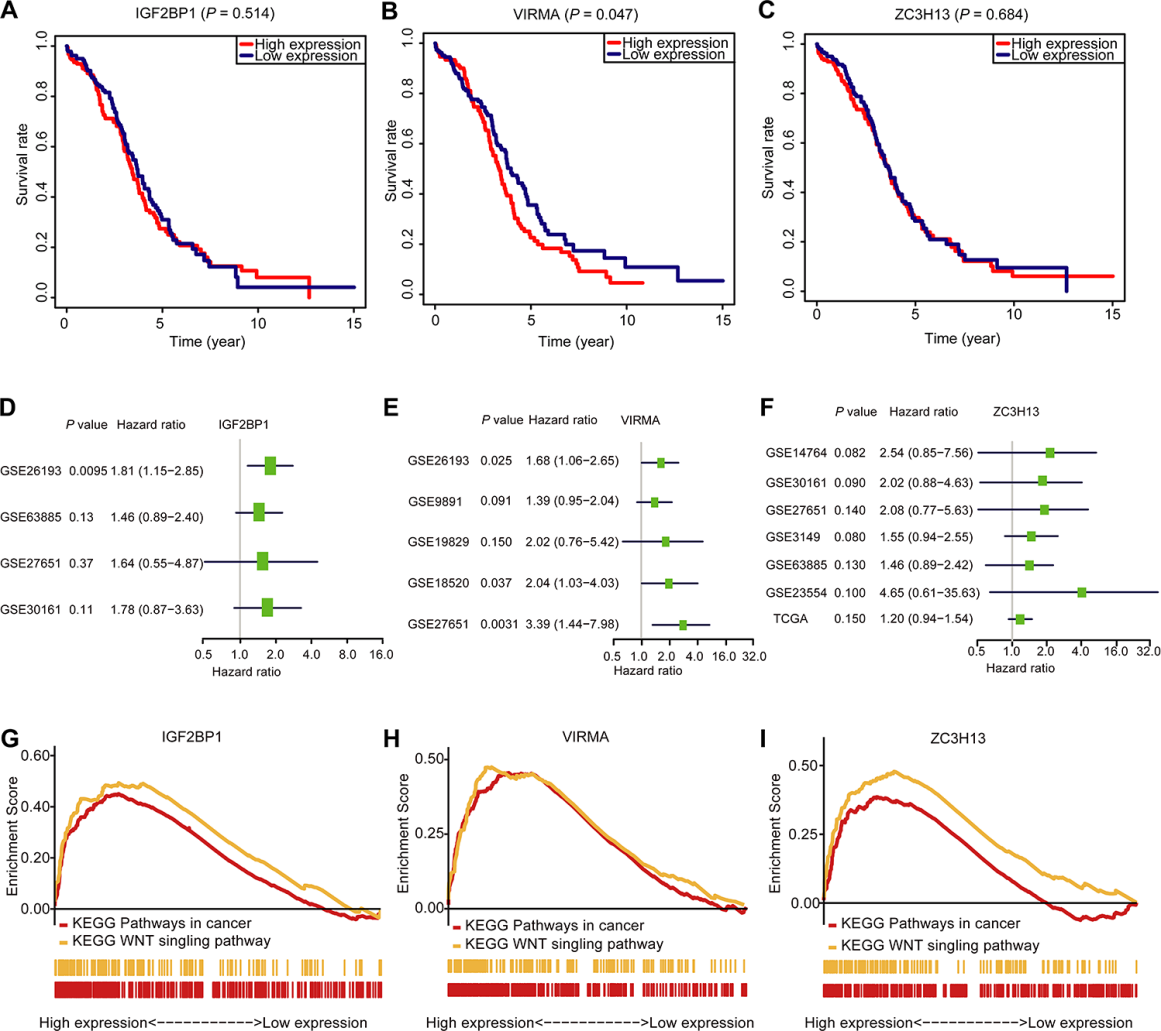
**The OS analysis, GSEA analysis, and validation in the GEO database of the three selected m^6^A RNA methylation regulators.** (**A**–**C**) OS survival curve of OC patients based on the three selected m^6^A RNA methylation regulators levels. (**D**–**F**) The validation of the three selected m^6^A RNA methylation regulators using the GEO database through univariate analysis. (**G**–**I**) Enrichment of genes in the Kyoto Encyclopedia of Genes and Genomes (KEGG) different pathways by GSEA.

To figure out the prognostic importance of each gene of the signature composed of IGF2BP1, VIRMA, and ZC3H13, the OS of patients with a high expression level of any gene of the signature was compared to that of patients with low expression. We noticed that OC patients with high VIRMA expression had a shorter median OS than those with low expression (*P* < 0.05, Figure 6B). Unexpectedly, for IGF2BP1 and ZC3H13, the OS rate of patients didn’t associate with their expression levels (Figure 6A and 6C). However, through univariate analysis, the association between the risk signature genes developed in this study can be validated using data derived from a different cohort downloaded from the Gene Expression Omnibus (GEO) database (Figure 6D–6F). This result further confirmed the efficiency of the risk signature to predict prognosis developed in this study. Using the Gene Set Enrichment Analysis (GSEA), we found that IGF2BP1, VIRMA, and ZC3H13 are all associated with pathways in cancer and WNT signaling pathway (Figure 6G–6I, Supplementary Table 1).

## DISCUSSION

Previous studies have pointed out that up- or down-regulation of specific RNA m^6^A methylation regulators are associated with the oncogenesis of many different tumors. Additionally, the same m^6^A methylation regulators might have different functions in different tumors. At present, the main work has been devoted to the study of the mechanism of m^6^A in promoting tumorigenesis [23–28].

OC is one of the deadliest gynecological malignancies. Most patients have stage III~IV at the moment of diagnosis, and the prognosis is poor [29, 30]. Classical epigenetics, restricted to DNA or protein modification, plays critical roles in OC initiation, malignant progression, and prognosis [31–33]. In our study, we found that the expression of another layer of epigenetic regulators, namely m^6^A RNA methylation, is also firmly associated with the malignancy and prognosis of OC.

Here, we firstly analyzed the expression of m^6^A RNA methylation regulators in OC and normal tissues and the relationship between their expression. Then we applied the consensus clustering to divide all OC samples into three clusters and analyzed the expression of m^6^A RNA methylation regulators and different clinicopathological variables according to the clustering. However, the results of PCA did not show a clear distinction among cluster1, 2 and 3, and there was almost no significant difference in terms of prognosis and clinical case characteristics among the three groups. The possible reasons could be that the sample size is not big enough to reflect the difference, or the clustering algorithm is not sensitive enough for these data.

Next, we explored the prognostic value of each m^6^A RNA methylation regulators and developed a risk signature applying three chose m^6^A RNA methylation regulators, IGF2BP1, VIRMA and ZC3H13, which are selected by the Cox univariate analysis and LASSO Cox regression analysis. Based on this signature, we established a nomogram that assimilated the three selected regulators associated with OC prognosis and used univariate analysis and multivariate analysis to assess the prognostic value of the three m^6^A RNA methylation regulators. At last, the OS analysis, GSEA analysis were applied to data collected from a different OC cohort to validate the prognostic value of the three selected m^6^A RNA methylation regulators. This work provided a different biomarker other than the tumor stage for predicting the prognosis of OC.

For the three selected regulators, IGF2BP1, VIRMA, and ZC3H13, the GSEA study result indicated that they were also all correlated with pathways in cancer and WNT signaling pathways. Corresponding to the results in the GSEA, Felicite K. Noubissi et al. [34] indicated that IGF2BP1 plays a central role in carcinoma development. They also mentioned that IGF2BP1 was a direct target of the WNT/β-catenin signaling. Consistently, another analysis using TCGA data indicated that the expression of IGF2BP1 was negatively associated with survival in prostate cancer [35]. Compared with these regulators, there are relatively few studies on the signal pathway mediated by VIRMA and ZC3H13. More relevant studies are needed in the future to reveal the signaling pathways that these genes are involved in and their physiological and pathological mechanisms both in vitro and in vivo.

Another interesting finding is, when we analyzed the experimentally determined protein-protein interactions between these three selected m^6^A RNA methylation regulators and the other proteins (Supplementary Figure 1B), we found ZC3H13 interacted with CDKs family, POLR2 family and MED complex family closely, which is consistent with the result in Supplementary Figure 1A. In mammalian cells, cyclin-dependent kinases (CDKs) control critical cell cycle checkpoints and RNA polymerase II-dependent transcriptional events in response to extracellular and intracellular signals leading to proliferation [36, 37]. Mediator (MED) is a large multiprotein complex conserved in all eukaryotes that plays an essential role in transcriptional regulation. The mediator comprises 30 subunits in humans that form three main modules and a separable four-subunit kinase module [38]. The mediator complex interacts with DNA-binding gene-specific transcription factors to modulate transcription by RNA polymerase II [39]. This suggested that ZC3H13 was closely involved in the transcription process, which was consistent with the known function of the m^6^A RNA methylation regulators.

As for the inconsistency between IGF2BP1 and VIRMA protein levels and mRNA levels (Figure 2A and Supplementary Figure 8A, 8B), this is a phenomenon that has been observed before in studying the expression of other genes. Yanqing Liu et al. used Pearson’s correlation analysis of scatter plots to reveal an inconsistent relationship between HuR protein levels and mRNA levels in colorectal cancer tissues, and pointed out that the inconsistency between HuR protein and mRNA levels indicated that some post-transcriptional gene regulation mechanisms were involved in the control of HuR expression [40]. Pernilla Israelsson et al. evaluated the relative mRNA expression and the corresponding protein expression of the cytokines IL-6, IL-8, TNF-α and TNF-β/LTA at seven consecutive time points in the kinetic experiments with ovarian cancer cell lines OVCAR-3 and SKOV-3 and compared with T cell line Jurkat, which served as a control [41]. They also observed inconsistency between protein and mRNA expression. In addition, Xinyu Ren et al. found that PD1/PDL1 mRNA and protein expressions were inconsistent too in triple-negative breast cancer [42]. In short, the inconsistency of protein and mRNA expression levels observed in this study is not a rare phenomenon, the specific details of which require more experiments and analysis.

**Figure 7:**
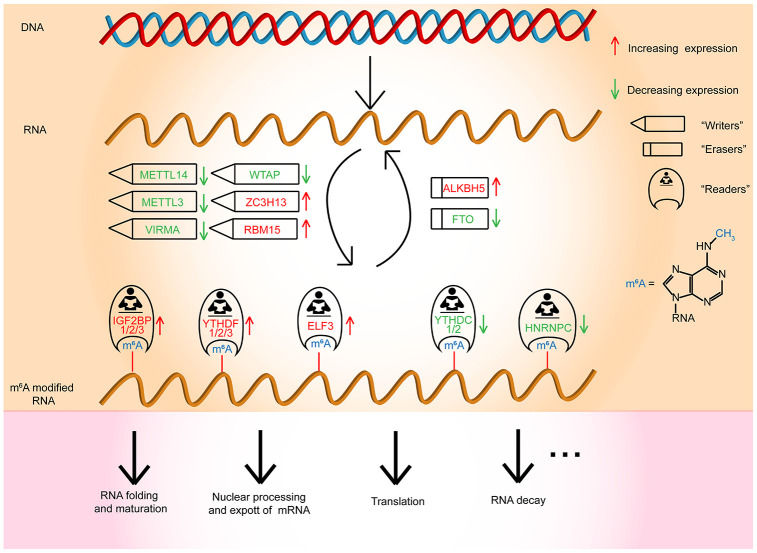
**Outline for the expression changes, mechanism, and potential functions of m^6^A RNA methylation regulators in OC.**

In conclusion, our studies comprehensively manifested the expression and prognostic value of m^6^A RNA methylation regulators in OC (Figure 7). The three selected m^6^A RNA methylation regulators, which were OC prognosis-associated factors, were also enriched in the biological processes and signaling pathways that drive the malignant progression of OC. In brief, our study provides novel markers for evaluating OC prognosis and furnishes significant proof for future research on the role of RNA m^6^A methylation in OC.

## MATERIALS AND METHODS

### Datasets

In March 2019, we obtained the RNA-seq transcriptome data of 379 OC patients and the corresponding clinicopathological information of 587 OC patients from the TCGA database (http://cancergenome.nih.gov/) and obtained the RNA-seq transcriptome data of 88 normal human ovarian tissues from GTEx database (https://www.gtexportal.org/home/datasets). For the RNA-seq data, TCGA samples (n = 379) were normalized by fragment per kilobase of exon model per Million (FPKM, namely Fragment Per Kilobase Million, which is defined in this way [43]).

### Selection and differential expression analysis of m^6^A RNA methylation regulators

We collected a list of 18 m^6^A RNA methylation regulators from published literature [44, 45]. Next, we systematically contrasted the expression of these m^6^A RNA methylation regulators in ovarian with different clinicopathological characters. All data were processed using the R software (version 3.4.0). The “*limma*” package was used for identifying DEGs between the OC samples and matched non-cancerous samples. The screening conditions for the differential genes were: *P* < 0.05(“*”), *P* < 0.01(“**”), *P* < 0.001(“***”). Heat maps of differential genes were drawn using the R-package, “*pheatmap*”.

### Bioinformatic analysis

To evaluate the prognostic value of m^6^A RNA methylation regulators, we executed univariate Cox regression analyses of their expression in the TCGA dataset, from which we selected three regulators virtually associated with survival (*P* < 0.1), which we chose for further functional research and development of a potential risk signature with the LASSO Cox regression algorithm [46, 47]. Finally, three regulators and their coefficients were decided by the minimum criteria, choosing the best penalty parameter λ associated with the TGGA datasets. The risk score for the signature was counted applying the formula:

$$Risks\;core=\sum_{i=1}^{n}Codfi\;*x_{i}{}^{\left[48\right]},$$

where Coefi is the coefficient, and x_i_ is the z-score transformed relative expression value of each selected regulator. This formula was applied to count a risk score for each patient in TGGA datasets. The high-risk subtype (samples with the risk score higher than 5) and the low-risk subtype (samples with risk score lower than 5) were defined in OC cases based on the risk score of its tumor samples. The ROC analysis was used for testing if the survival prediction is sensitive and specific based on the risk score.

### PPI network construction of m^6^A RNA methylation regulators

Protein-protein interactions (PPI) analysis was conducted to reveal the molecular mechanisms of a list of 18 m^6^A RNA methylation regulators in ovarian cancer. We utilized the Search Tool for the Retrieval of Interacting Genes (STRING) protein database 11.0 (http://string-db.org/) to construct the PPI networks. An interaction score > 0.4 was regarded as the cut-off criterion.

### Gene set enrichment analysis (GSEA) of the three selected m^6^A RNA methylation regulators

Gene Set Enrichment Analysis (GSEA) of prognosis-related MeDEGs was performed using GSEA 3.0 software with gene set c2 (cp.kegg.v.6.2.symbols.gmt). High throughput RNA expression of 379 ovarian cancer genes from TCGA was utilized as the dataset. Each sample was defined as either “H” or “L”, depending on whether it was greater than the median mRNA expression value of prognosis-related DEGs or not. The number and type of permutations were set at “1000” and “phenotype,” respectively. An enrichment score >0.4 and *P* < 0.05 were regarded as statistically significant.

### Tumor subgroup gene expression and survival analyses

UALCAN (http://ualcan.path.uab.edu/index.html) is a portal for facilitating tumor subgroup gene expression and survival analyses for analyzing cancer OMICS data. Using TCGA transcriptome and clinical patient data, compare across different tumor subgroups as defined by the patient’s age and tumor grade through the expression level of the gene. Finally, the primary tumor samples were categorized using OC patient clinical data, and boxplots were generated of the expression level of each gene across various subgroups.

### Survival Analysis of IGF2BP1, VIRMA and ZC3H13

Download the datum matrix containing all ovarian patients’ prognosis information from the GEO database. The overall survival analysis was conducted using the only patient with survival data and gene expression data from RNA-seq. For each gene, a tab-separated input file was created with columns for TCGA sample id, Time (days_to_death), Status (Alive or Dead), and Expression level. Samples were categorized into two groups according to the level of gene expression: (1) High expression (with values above the average) and (2) Low expression (with values below the average). The data was processed by R packages *“survival”*. Kaplan-Meier curves were drawn to demonstrate the relationship between the patient’s overall survival and gene expression levels of m^6^A RNA methylation regulators. The relationship was tested by the log-rank test.

### Validation of the IGF2BP1, VIRMA and ZC3H13

To determine the robustness of this model, we used the same coefficients from the training set to validate in the validation sets including GSE30161 (n = 58), GSE9891 (n = 285), GSE63885 (n = 101), GSE19829 (n = 70), GSE18520 (n = 63), GSE26193 (n = 107), GSE27651 (n = 49), GSE14764 (n = 80), GSE3149 (n = 153), GSE23554 (n = 28), and TCGA (n = 427) dataset. We used multivariate Cox proportional analysis to determine a panel of prognostic genes. The calculation of the patient’s risk score in the training set was performed according to the formulate obtained from the multivariate Cox proportional model. The forest plots were used to display the multivariable Cox results, including all the above variables. The *“forestplot”* R packages were used to draw forest plots.

### Independent prognostic analysis

The univariate and multivariable Cox regression analyses were utilized to access the prognostic value of the risk score generated from the multivariate model. The demographics and clinical information, including age and grade, were used for model correction.

### Statistical analysis

One-way ANOVA was applied to contrast the expression levels of m^6^A RNA methylation regulators in ovaries with normal patients group (GTEx datasets) and tumor patients group (TCGA datasets), and t-tests were applied to contrast the expression levels in OC patients for grade, age and survival status.

Patients were grouped into three clusters by consensus expression of m^6^A RNA methylation regulators or were separated into low-risk and high-risk groups applying the median risk score (came from the risk signature) as the cut-off value. Chi-square tests were applied to contrast the distribution of patients’ age, survival status and the grade between the two risk groups.

To contrast the risk scores of the signature for ovaries with different clinicopathologic, a one-way ANOVA or t-test was conducted to contrast the risk scores in patients divided by clinical or molecular-pathological features. Univariate and multivariate Cox regression analyses were conducted to evaluate the prognostic value of the risk score and various clinical and molecular-pathological features. The prediction efficiency of the risk signature was tested with the ROC curve.

The Kaplan-Meier method with a two-sided log-rank test was applied to contrast the OS of the patients in the cluster 1/2/3 groups or in the low- and high-risk groups. All statistical analyses were executed utilizing R v3.4.1 (https://www.r-project.org/) and Prism 8 (GraphPad Software Inc., La Jolla, CA).

## Supplementary Material

Supplementary Figures

Supplementary Table 1
