# Supplementary material for: A newly defined risk signature, consisting of three m6A RNA methylation regulators, predicts the prognosis of ovarian cancer
Source: Aging (Albany NY). 2020 Sep 20;12(18):18453–75. doi: 10.18632/aging.103811 (PMC7585096; doi:10.18632/aging.103811)
Supplement: Supplementary Figures [file aging-12-103811-s002..pdf]

SUPPLEMENTARY FIGURES

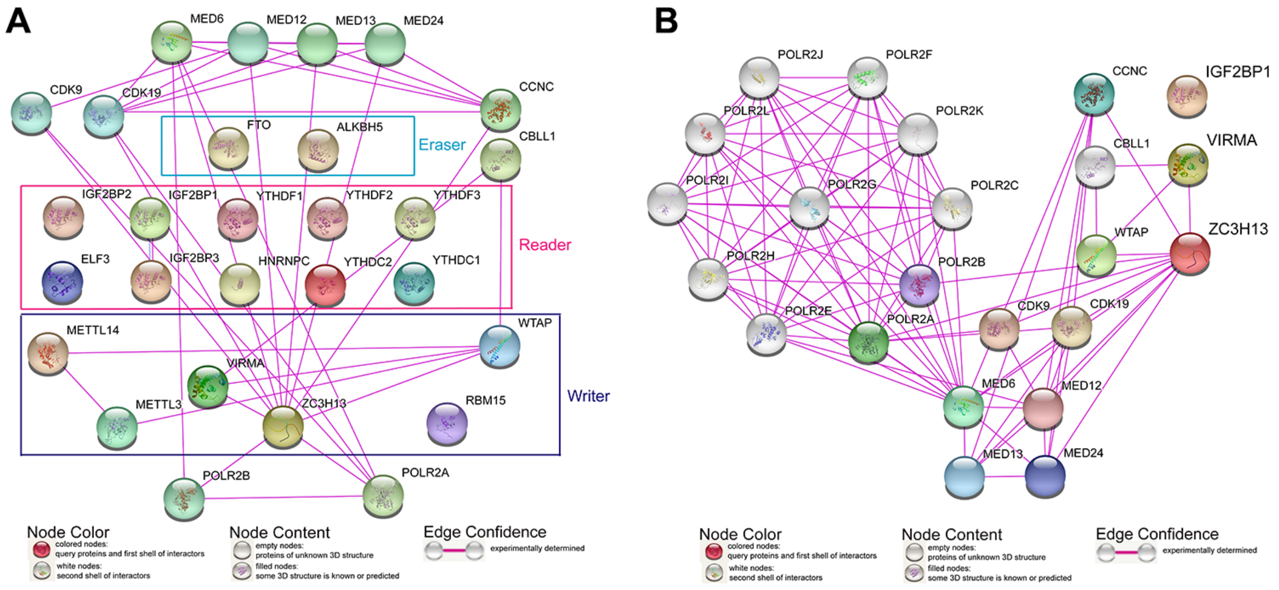

Supplementary Figure 1. The protein-protein interaction network analysis. (**A**) The protein-protein interaction network analysis between the 18 m^{6}A regulators and the other proteins. (**B**) The protein-protein interaction network analysis between these three selected m^{6}A RNA methylation regulators and the other proteins.

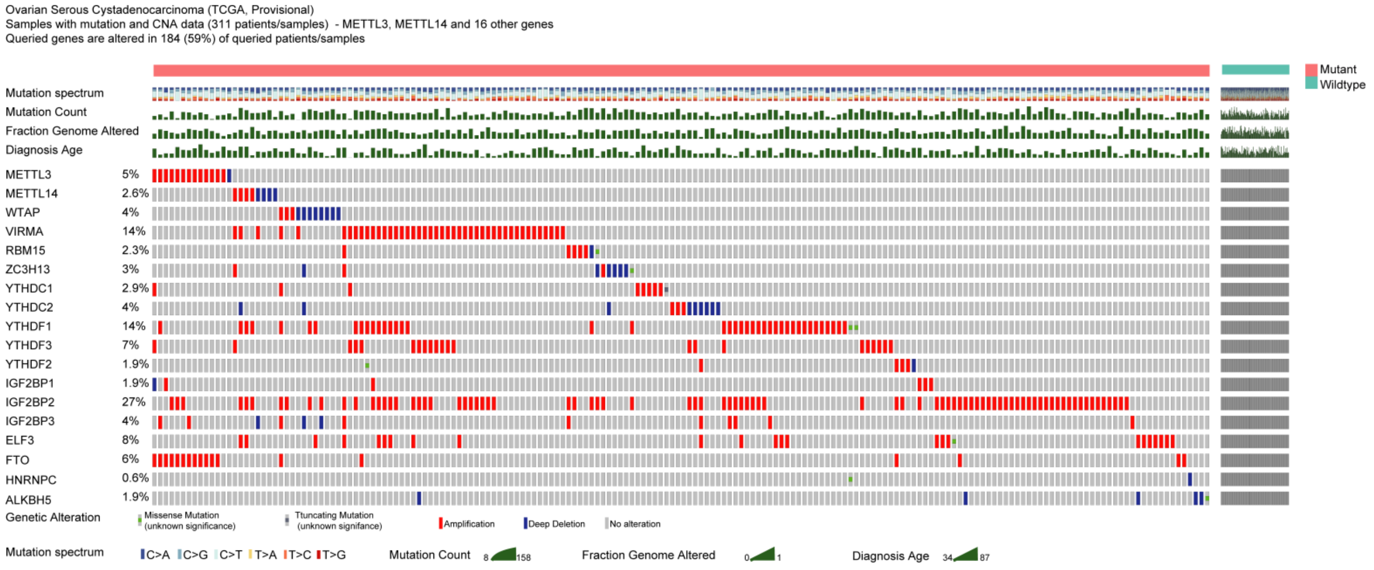

Supplementary Figure 2. Genetic changes of RNA m^{6}A regulators in the TCGA dataset. Genetic changes of the 18 m^{6}A RNA methylation regulators in the 379 OC from the TCGA dataset.

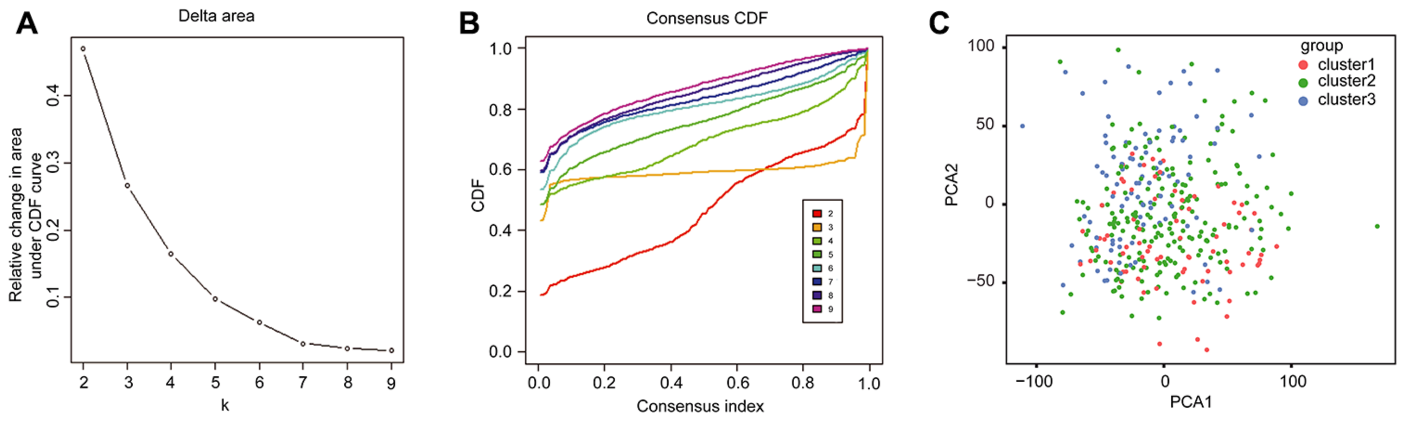

Supplementary Figure 3. Identification of consensus clusters by m^{6}A RNA methylation regulators and PCA. (**A**) Comparative change in area under cumulative distribution function (CDF) curve for k = 2 to 10. (**B**) Consensus clustering CDF for k = 2 to 10. (**C**) PCA of the total RNA expression profile.

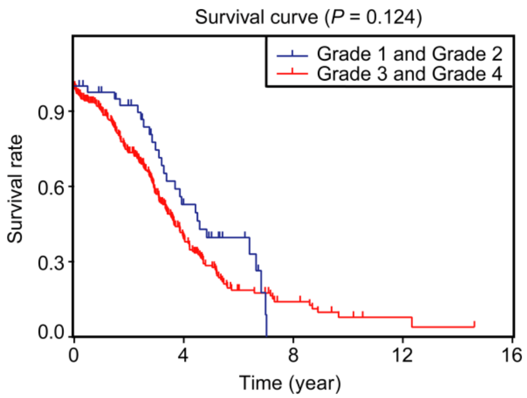

Supplementary Figure 4. OS of OC patients with different tumor grades. Kaplan-Meier OS curves for patients in the TCGA datasets depended on grade.

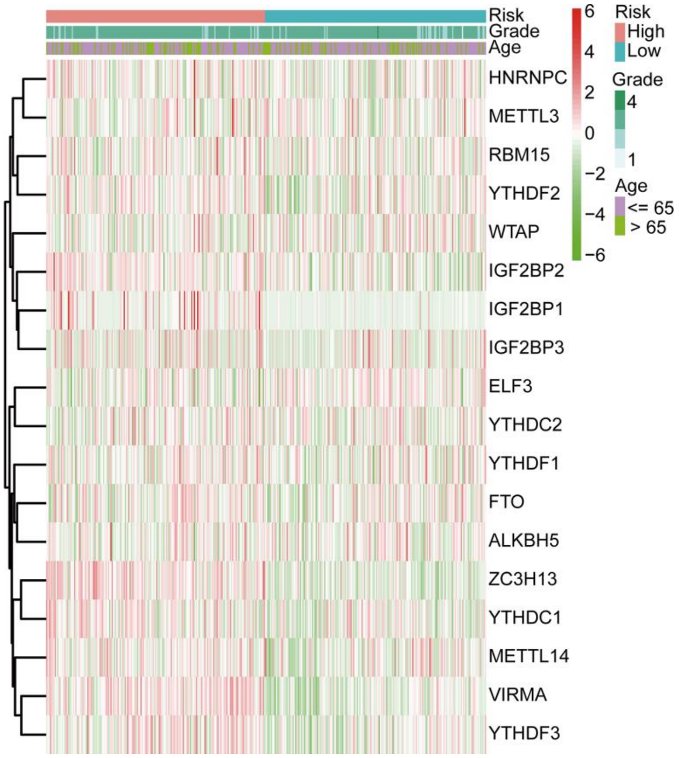

Supplementary Figure 5. Heatmap of the 18 m^{6}A RNA methylation regulators. Heatmap and clinicopathologic features of the two clusters (high risk and low risk) defined by the 18 m^{6}A RNA methylation regulators' risk score.

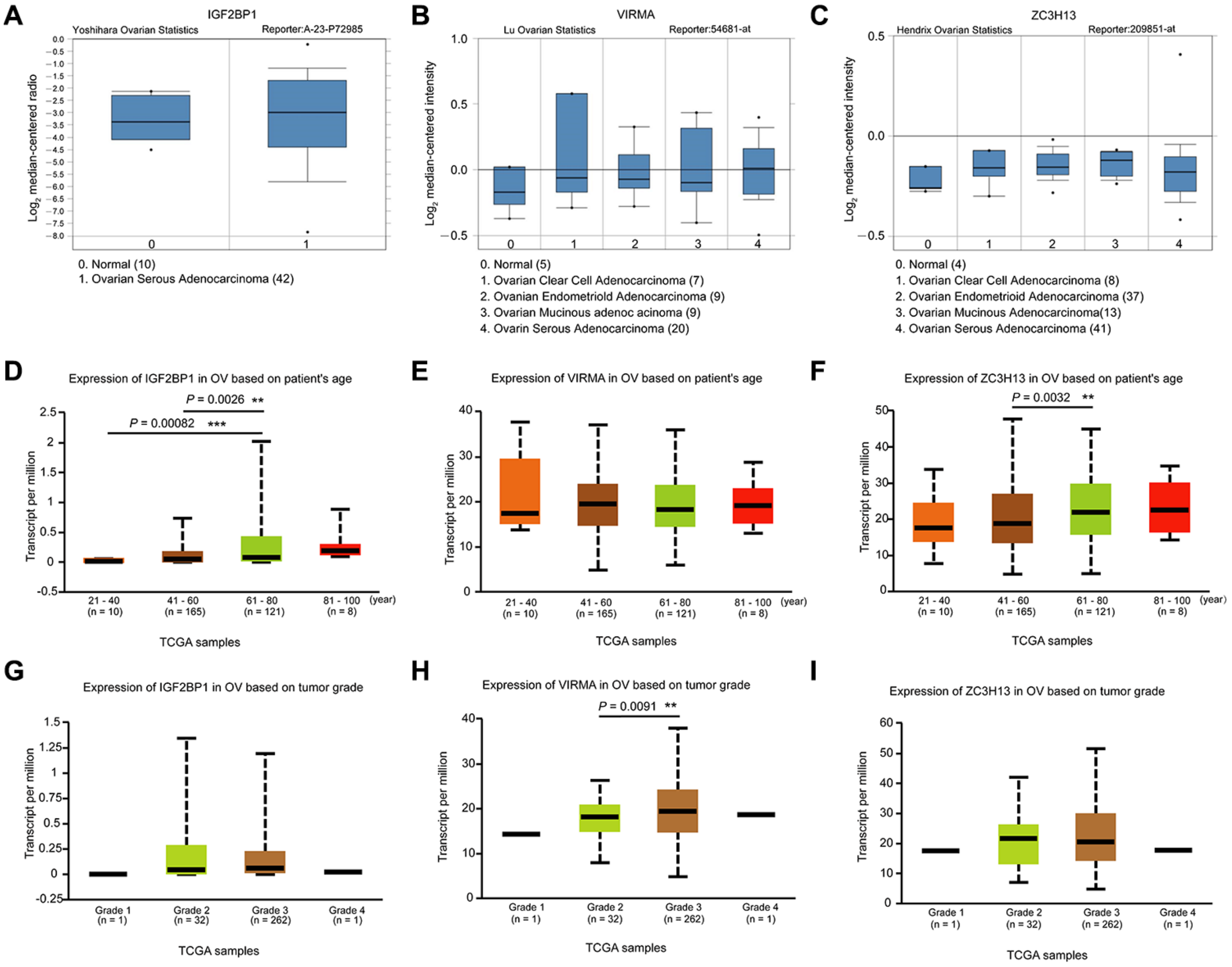

Supplementary Figure 6. Expression of IGF2BP1, VIRMA and ZC3H13 in OC patients with different types, ages and grades. (**A**) Expression of IGF2BP1 in different ovarian cancer in the Oncomine database. (**B**) Expression of VIRMA in different ovarian cancer in the Oncomine database. (**C**) Expression of ZC3H13 in different ovarian cancer in the Oncomine database. (**D**) Expression of IGF2BP1 in OV based on the patient's age. (**E**) Expression of VIRMA in OV based on patient's age. (**F**) Expression of ZC3H13 in OV based on the patient's age. (**G**) Expression of IGF2BP1 in OV based on tumor grade. (**H**) Expression of VIRMA in OV based on tumor grade. (**I**) Expression of ZC3H13 in OV based on tumor grade. OV: Ovarian serous cystadenocarcinoma

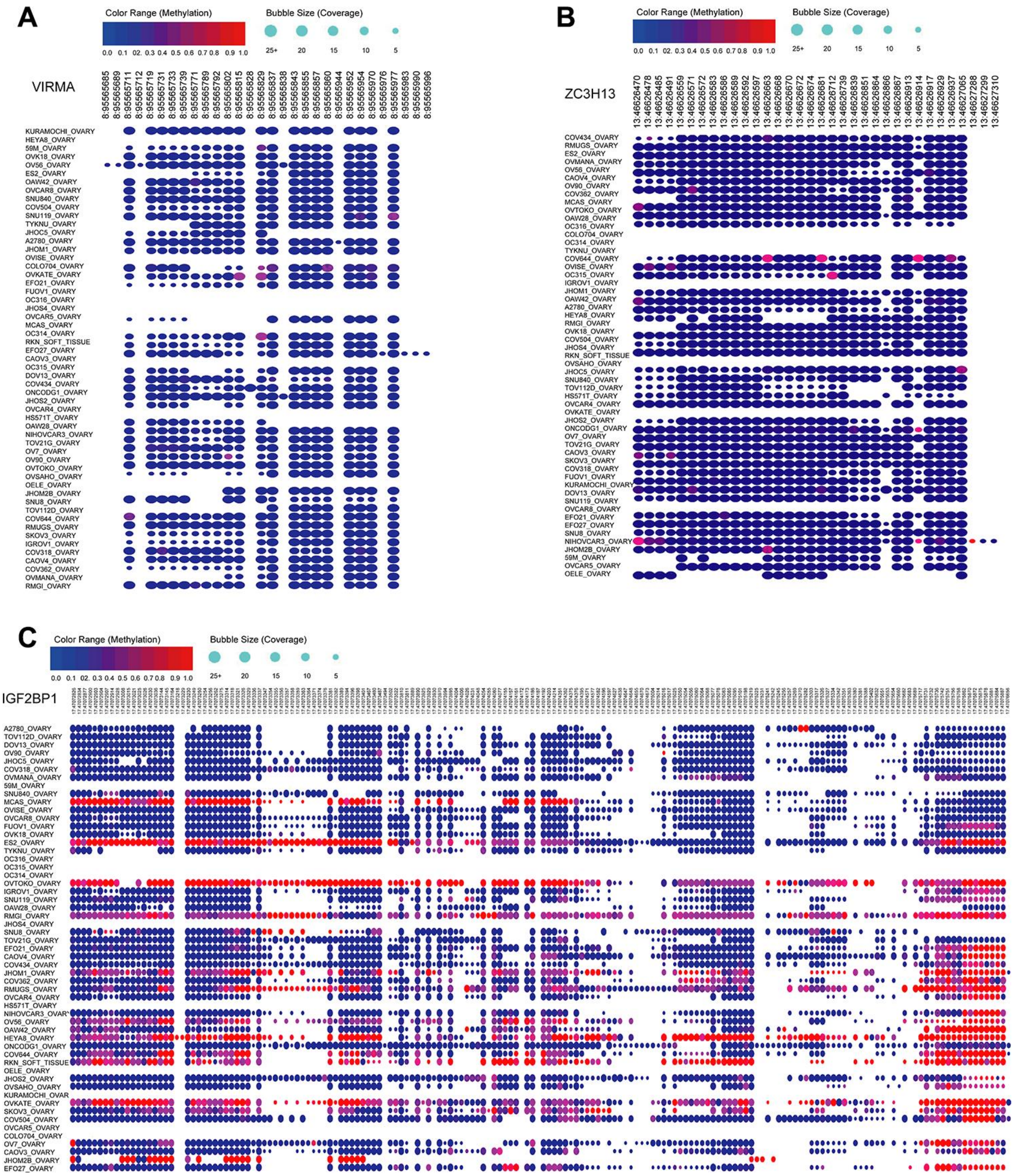

Supplementary Figure 7. Mutation data of three selected m^{6}A RNA methylation regulators in different OC cell lines based on the Cancer Cell Line Encyclopedia (CCLE) database. (**A**) Mutation sites of VIRMA in different OC cell lines. (**B**) Mutation sites of ZC3H13 in different OC cell lines. (**C**) Mutation sites of IGF2BP1 in different OC cell lines.

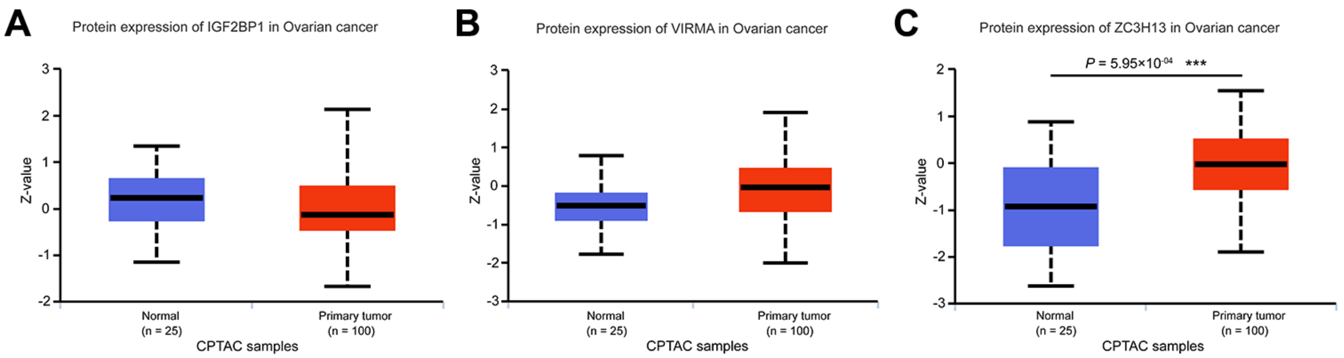

Supplementary Figure 8. Protein expression of IGF2BP1, VIRMA and ZC3H13 in OC patients in the CPTAC samples. (**A**) Protein expression of IGF2BP1in OC patients. (**B**) Protein expression of VIRMA in OC patients. (**C**) Protein expression of ZC3H13 in OC patients.
