# Supplementary material for: A newly defined risk signature, consisting of three m6A RNA methylation regulators, predicts the prognosis of ovarian cancer
Source: Aging (Albany NY). 2020 Sep 20;12(18):18453–75. doi: 10.18632/aging.103811 (PMC7585096; doi:10.18632/aging.103811)
Supplement: Supplementary Table 1 [file aging-12-103811-s001..pdf]

SUPPLEMENTARY TABLE

Supplementary Table 1. Specific Information on the GSEA analysis of the three selected m^6A RNA methylation regulators.

m^6A methylation regulators	Pathways	NES	normalized P
IGF2BP1	Pathways in cancer	1.83	0.006
	WNT signaling pathway	1.91	0.004
VIRMA	Pathways in cancer	1.88	0.002
	WNT signaling pathway	1.86	0.004
ZC3H13	Pathways in cancer	1.59	0.037
	WNT signaling pathway	1.91	0.004

Pathways associated with IGF2BP1, VIRMA and ZC3H13.
